# The association between gynecological complaints and the uterine sonographic features in women with a history of cesarean section

**DOI:** 10.1007/s00404-024-07526-x

**Published:** 2024-05-02

**Authors:** Helen Kellner, Alex Horky, Frank Louwen, Franz Bahlmann, Ammar Al Naimi

**Affiliations:** 1 Department of Obstetrics and Gynecology, Buergerhospital – Dr. Senckenberg Foundation, Nibelungenallee 37-41, D-60318 Frankfurt Am Main, Hessen Germany; 2 Department of Obstetrics and Prenatal Medicine, University Hospital of the Goethe University of Frankfurt, Hessen, Germany

**Keywords:** Cesarean section, Sonography, Bleeding disorder, Dysmenorrhea, Cesarean scar

## Abstract

**Purpose:**

The aim of this study is to investigate the association between post-cesarean sonographic uterine measures, dysmenorrhea, and bleeding disorders.

**Methods:**

This is a cross-sectional study where 500 women with a history of only one cesarean section (CS) were recruited. A transvaginal transducer, GE RIC6-12-D was used for the acquisition of volumetric datasets 18 ± 7 months postpartum. Uterine length (UL), cervical length (CL), niche length (L), niche depth (D), niche width (W), fibrosis length (FL), fibrosis depth (FD), residual myometrial thickness (RMT), endometrial thickness (EM), scar to internal os distance (SO), anterior myometrial thickness superior (sAMT) and inferior (iAMT) to the scar, and the posterior myometrial thickness opposite the scar (PMT), superior (sPMT), and inferior to it (iPMT) were measured. Logistic regression with odds ratios (OR), 95% confidence intervals (CI) and ROC curves were utilized.

**Results:**

The proportion of patients with incident post-cesarean bleeding disorders and dysmenorrhoea was 36% (CI 32%, 40%) and 17% (CI 14%, 21%) respectively. Univariate logistic regression showed that only UL was associated with bleeding disorders [OR 1.04 (CI 1.01,10.7) *p *value 0.005], whereas dysmenorrhea was associated with RMT [OR 0.82 (CI 0.71,0.95) *p* value 0.008], SO [OR 0.91 (CI 0.86,0.98) *p *value 0.01], and RMT ratio [OR 0.98 (CI 0.97,0.99) *p* value 0.03]. Multivariate logistic regression for dysmenorrhoea including SO and RMT remains statistically significant with *p *values <0.05 and area under the curve of 0.66.

**Conclusion:**

There is an association between sonographic appearance of CS scars and dysmenorrhoea. Nevertheless, the association is weak and other biological post-cesarean characteristics should be explored as potential causes.

## What does this study adds to the clinical work

**Table:** 

Despite the association between the sonographic appearance of cesarean scars and dysmenorrhoea and bleeding disorders, the utility of ultrasound in explaining these complaints is clinically limited.	

## Introduction

Cesarean section (CS) is one of the most frequently performed surgeries worldwide. The CS rate in Germany doubled in the last 30 years to reach an all-time high of about 30.9% according to a 2023 press release from the Federal Statistical Office of Germany [1]. Many developed countries have CS rates of 30% or more. Despite the fact that CSs are routine and often performed procedures, they increase the risk of adverse maternal outcomes in comparison to vaginal delivery [2]. A history of CS is associated with a serious long-term risk of abnormal uterine bleeding, chronical pelvic pain, dysmenorrhea and dyspareunia [3], but the pathogenesis behind these symptoms is not fully understood. The uterine wall incision during CS could heal entirely or leave a defect called a “niche”. The prevalence of a niche greatly varies depending on the selection of the study population (random, symptomatic, or non-symptomatic), the utilized diagnostic tools (transvaginal sonography, contrast-enhanced sonohysterography, or hysteroscopy), the study sample size, and the utilized definition of the diagnostic criteria. Sonographic prevalence ranges from 24 to 70%, and the characteristics and the size of niches are associated with possible gynecological complaints [4, 5]. Menstrual debris is suspected to collect in the uterine wall defects (niche) and delayed emptying of this debris can then cause postmenstrual spotting and intermenstrual bleeding. Moreover, dysfunctional uterine contraction in an attempt to clear the niche of any debris could be the reason for the pelvic pain [6]. Furthermore, a previous CS increases the risk of abnormally invasive placenta, extrauterine pregnancy, uterine rupture, and hysterectomy in a subsequent pregnancy [2]. A better understanding of cesarean scars and their outcomes is crucial to allowing a profound consultation and treatment of women who show any of these symptoms.

Especially after Delphi-based guidelines to standardize the sonographic measurement of niches were published by Jordans et al. in 2019 [7], ultrasound is considered to be the gold standard imaging technique for assessing the condition of the uterine wall and any possible scaring after CS [8]. In addition to measuring length, width, and depth of the niche, other measurements like the residual myometrial thickness (RMT) and the distance between the niche and the external os ought to be taken into consideration as well [9].

The aim of this work is to assess the association between the standardized uterine sonographic measurements after a CS and incident dysmenorrhea and dysfunctional bleeding. Establishing such an association could be beneficial in building predictive models of gynecological complications for women with a history of CS using sonographic features.

## Methods

**Fig. 1:**
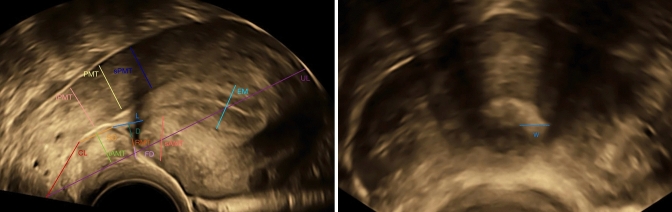
Transvaginal ultrasound of a post-cesarean uterus with the measures of the study: uterine length (UL), cervical length (CL), niche length (L), niche depth (D), niche width (W), RMT, endometrial thickness (EM), scar to internal os distance (SO), anterior myometrial thickness superior (sAMT) and inferior (iAMT) to the scar and the posterior myometrial thickness opposite the scar (PMT), and superior (sPMT) and inferior to it (iPMT)

The data utilized in this cross-sectional study were collected within the BSUM study. The BSUM study is a prospective observational multicenter clinical study that included consenting patients over the age of 18 with a history of only one CS and open family planning. Excluded from the study were patients with more than one CS, a history of vertical hysterotomy or additional uterine surgeries as well as completed family planning. The study was approved by the Ethical Committee of the Hessen Regional Medical Council (Reg. No. 2019-1138-evBO). A total of 500 women were recruited and the patients were examined with transvaginal ultrasound in a lithotomy position and with an empty bladder. A Voluson E10 with a 5–13 MHz GE RIC6-12-D microconvex transvaginal transducer was used for the sonographic evaluation. The timing of examination was at least one year postpartum with a mean of 18 ± 7 months. Volumetric three-dimensional data of the uterus were acquired and analyzed offline for measuring uterine length (UL), cervical length (CL), niche length (L), niche depth (D), niche width (W), residual myometrial thickness (RMT), endometrial thickness (EM), scar to internal os distance (SO), anterior myometrial thickness superior (sAMT) and inferior (iAMT) to the scar and the posterior myometrial thickness opposite the scar (PMT), and superior (sPMT) and inferior to it (iPMT) as per the protocol of the BSUM study [10] and shown in Fig. 1.

**Fig. 2:**
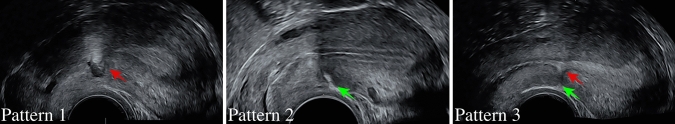
Transvaginal ultrasound showing the typical appearance of the niche (*red arrow*) and fibrosis (*green arrow*) among the three patterns of the uterine scar (color figure online)

The largest depth of external denting (fibrosis) was measured as (FD) when fibrosis was identified and the uterine scar was classified into three patterns as shown in Fig. 2; pattern 1 showed scars with only a niche, pattern 2 scars with only fibrosis, and pattern 3 scars with both niches and fibrosis.

Moreover, RMT ratio which is the percentage of RMT to the pre-CS anterior wall thickness was calculated according to the formula ‘RMT ratio = RMT × 100/(D + RMT + FD)’ and the volume of the niche was calculated (V = L × D × W). Furthermore, the demographic characteristics and the outcome measures of newly incident dysfunctional uterine bleeding and dysmenorrhea were ascertained at the time of the examination by interviewing the patients and reviewing their medical charts. The outcome measures were based on the standardized nomenclature of the American College of Obstetricians and Gynecologists (ACOG). Dysmenorrhea was defined as postsurgical unusually painful menstruation, and dysfunctional uterine bleeding was defined as menstrual flow with volume, duration, frequency, or regularity outside of the individual’s pre-surgical normal [11, 12]. The association between the different sonographic measures as independent variables and the two main outcomes of dysmenorrhea and dysfunctional bleeding as dependent variables was tested with univariate and multivariate logistic regressions, and receiver operating curves were utilized for assessing the performance of the resulting models. The association results from the logistic regression are presented as odds ratios (OR) and 95% confidence intervals (CI) and all statistical analyses were performed with STATA (ver. 18, Texas, USA).

## Results

The mean maternal age at delivery was 35.5 ± 5.2 years and 196 (39.2%) of the CSs were elective. The base demographic characteristics of the study population are summarized in Table 1.

**Table 1:** Demographic data of the study cohort

Characteristic	Mean ± SD Median (IQR) Number (percentage %)
Maternal age (years)	35.5 ± 5.2
Gestational age at delivery (weeks)	38 ± 3.5
Elective cesarean section	196 (39.2%)
*Surgical indications*	
Fetal distress	125 (25%)
Obstructed labor	103 (20.6%)
Breech	85 (17%)
Choice	32 (6.4%)
IUGR	24 (4.8%)
Other	131(26.2%)
Cervical dilatation (cm)	3 (1–6)
Obesity	29 (5.8%)
Antepartal infection	41 (8.2%)
Diabetes	23 (4.6%)

*SD* standard deviation, *IQR* interquartile range, *IUGR* intrauterine growth restriction

The questionnaire revealed that 17% (CI 14%, 21%) of women experienced dysmenorrhea while 36% (CI 32%, 40%) of women expressed a struggle with abnormal uterine bleeding after CS. Histograms, quartile–quartile plots, measures of skewness and kurtosis, and Shapiro–Wilk test were utilized to test the assumptions of normality and the distribution of the sonographic findings is summarized in Table 2.

**Table 2:** Summary of the distribution for all sonographic measurements in mm

Sonographic finding	Mean ± SD Median (IQR) Number (percentage %)
Uterine length (UL)*	69.06 ± 9.36
Cervical length (CL)*	20.78 ± 3.7
Residual myometrial thickness (RMT)	5 (3.2–6.5)
Scar to internal os distance (SO)*	9.79 ± 5.57
Endometrial thickness (EM)	6.3 (4.2–9)
Anterior myometrial thickness superior (sAMT)*	10.44 ± 2.89
Anterior myometrial thickness inferior (iAMT)	8.8 (7.5–10.2)
Posterior myometrial thickness opposite the scar (PMT)	11.4 (9.7–13.1)
Posterior myometrial thickness superior to scar (sPMT)*	12.58 ± 3.17
Posterior myometrial thickness inferior to scar (iPMT)	10.6 (8.8–12.1)
Residual myometrial thickness ratio (RMT%)	57.21 (40.37–71.84)
Niche	405 (81%)
Fibrosis	235 (47%)
Volume of the niche	80.08 (30.09–206.83)
Pattern 0 (no scar)	41 (8.2%)
Pattern 1 (niche)	221 (44.2%)
Pattern 2 (fibrosis)	53 (10.6%)
Pattern 3 (both niche and fibrosis)	185 (37%)

*SD* standard deviation, *IQR* interquartile range

The variables for which the assumptions of normality hold are marked with *

Univariate logistic regression was utilized to show the association between dysmenorrhea as well as bleeding disorders and each sonographic measure as portrayed in Table 3. There was an association between bleeding disorders and UL only [OR 1.04 (CI 1.01, 1.07) *p* value 0.005]. Dysmenorrhea, on the other hand, showed an association with RMT [OR 0.82 (CI 0.71, 0.95) *p* value 0.008], SO [OR 0.91 (CI 0.86, 0.98) *p* value 0.01], and RMT ratio [OR 0.98 (CI 0.97, 0.99) *p* value 0.03].

**Table 3:** Odds ratio with standard error and confidence interval as well as *p* value for the univariate logistic regression with all sonographic measurements

Sonographic finding	Bleeding disorders		Dysmenorrhea	
	Odds ratio (95% CI)	*p* value	Odds ratio (95% CI)	*p* value
UL	1.04 (1.01–1.07)	**0.005**	1.02 (0.99–1.06)	0.15
CL	1.05 (0.98–1.12)	0.18	0.96 (0.89–1.05)	0.40
RMT	0.93 (0.84–1.02)	0.12	0.82 (0.71–0.95)	**0.008**
SO	0.97 (0.92–1.01)	0.13	0.91 (0.86–0.98)	**0.01**
EM	1.03 (0.96–1.10)	0.42	1.07 (0.98–1.16)	0.11
sAMT	1.02 (0.94–1.11)	0.59	0.94 (0.84 1.05)	0.28
iAMT	0.96 (0.85–1.08)	0.46	0.92 (0.79–1.07)	0.27
PMT	0.99 (0.91–1.08)	0.89	0.98 (0.87–1.09)	0.70
sPMT	0.98 (0.91–1.06)	0.66	0.98 (0.89–1.09)	0.73
iPMT	0.99 (0.91–1.09)	0.9	1.01 (0.89–1.14)	0.91
RMT%	0.99 (0.98–1.00)	0.11	0.98 (0.97–0.99)	**0.03**
Niche	0.83 (0.45–1.56)	0.57	2.15 (0.80–5.73)	0.13
Fibrosis	1.04 (0.63–1.70)	0.88	0.65 (0.34–1.25)	0.19
Volume of the niche	1.00 (0.99–1.00)	0.18	1.00 (0.99–1.00)	0.19
Pattern 1 (niche)	1.71 (0.63–4.66)	0.29	6.55 (0.85–50.8)	0.07
Pattern 2 (fibrosis)	2.66 (0.83–8.53)	0.29	3.26 (0.34–31.3)	0.31
Pattern 3 (niche and fibrosis)	1.33 (0.48–3.70)	0.58	3.58 (0.45–28.7)	0.23

*CI* confidence interval, *UL* uterine length, *CL* cervical length, *RMT* residual myometrial thickness, *SO* scar to internal os distance, *EM* endometrial thickness, *sAMT* anterior myometrial thickness superior to the scar, *iAMT* anterior myometrial thickness inferior to the scar, *PMT* posterior myometrial thickness opposite the scar, *sPMT* posterior myometrial thickness superior to the scar, *iPMT* posterior myometrial thickness inferior to the scar, *RMT%* residual myometrial thickness ratio. Significant p-values are printed in bold

**Fig. 3:**
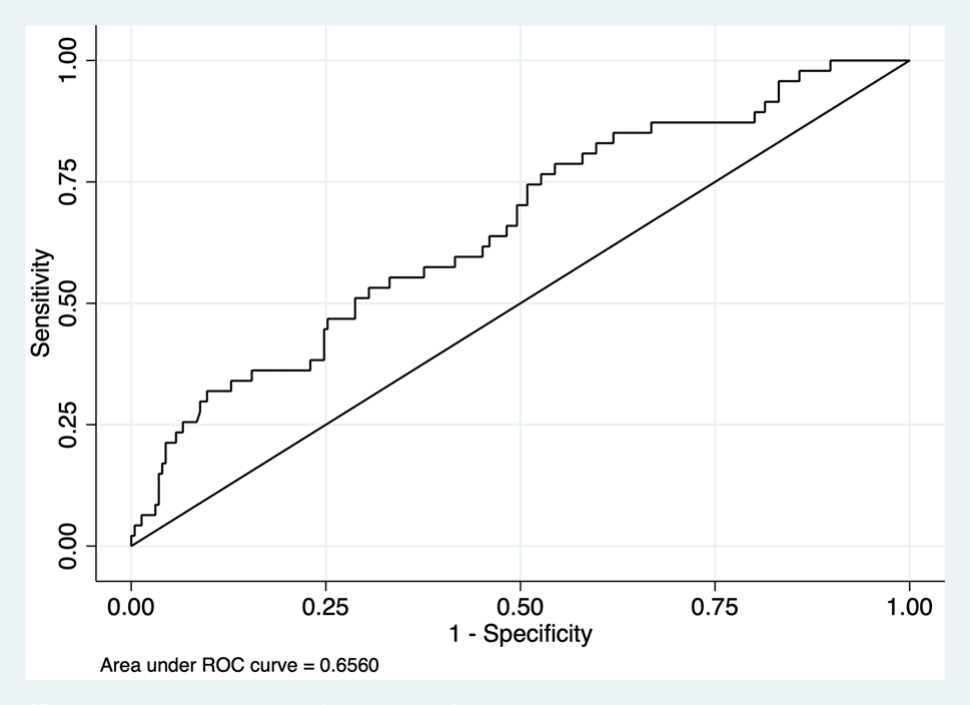
Receiver operating curve (ROC) analysis for dysmenorrhea predicted by residual myometrial thickness (RMT) and scar to internal os distance (SO)

Since dysmenorrhea showed a significant association with more than one variable, and multivariate logistic regression (using RMT and SO) and ROC analysis were performed. The *p* value of the multivariate logistic regression remained significant with *p* value <0.05 and the area under the ROC was 0.66 with pseudo *R*^2^ of 0.052 as shown in Fig. 3.

## Discussion

A recently published new nomenclature for cesarean scar disorder (CSDi) can be considered a milestone for the study of CS scars, niches, and post-cesarean complications. The modified Delphi procedure included 31 international niche experts who reached a consensus that CSDi should be used to describe post-cesarean niche-caused abnormal conditions [13]. Defining this diagnosis aimed to standardize the characteristics of this condition since no consistent definition for the combination of sonographic findings and clinical symptoms existed before. CSDi was specified as the presence of a niche, defined according to the guidelines by Jordans et al. [7], combined with either one primary or two secondary symptoms. The primary symptoms include postmenstrual spotting, dysmenorrhea, technical difficulties with catheter insertion during embryo transfer and secondary not otherwise explained infertility combined with intrauterine fluid, whereas dyspareunia, abnormal vaginal discharge, chronic pelvic pain, avoiding sexual intercourse, odor associated with abnormal blood loss, secondary unexplained infertility, infertility despite ART, negative self-image and discomfort during leisure activities are considered as secondary symptoms [13]. Nevertheless, it is essential to exclude other possible causes of the symptoms prior to diagnosing CSDi. The possible long-term outcomes of a CS can greatly impact women’s quality of life, and there is a consensus that many of these outcomes are niche-related but further distinctions remain missing.

The protective role of RMT against gynecological and obstetrical complications after a CS is considered to be essential. A prospective study of more than 300 patients showed that the ratio of niche depth to RMT could be utilized to predict uterine dehiscence in a prospective pregnancy [14]. Moreover, a cohort from Shanghai, with a sample size comparable to ours, showed that women who become symptomatic after CS have thinner RMT and are more likely to show a niche compared to asymptomatic women. They proposed that an RMT of about 5 mm is considered normal for asymptomatic women with satisfactory CS scar healing [15]. The importance of RMT was again emphasized in the niche sonographic evaluation guidelines [7]. Our findings, which showed a significant association between dysmenorrhea and RMT, confirm the importance of RMT during the sonographic CS scar evaluation. Furthermore, we found an association between SO and dysmenorrhea where an increase in SO is associated with a decreased risk of dysmenorrhea. This might indicate that women with unplanned CS, who tend to have smaller SO [16], could have an increased risk of dysmenorrhea compared to women with planned CS. Nevertheless, the resulting pseudo *R*^2^ of the multivariate logistic regression demonstrates that only about 5% of the dysmenorrhea cases can be explained by the sonographic findings. Our data highlight that the utility of ultrasound in predicting post-cesarean dysmenorrhea is extremely limited. About 95% of symptomatic patients do not exhibit the expected sonographic features. Therefore, we are unlikely to be solely relying on ultrasound for counseling these patients anytime soon. While there is a link between sonographic depiction of niches and dysmenorrhea, this association is weak such that it should not be seen as the sole explanation. Further biological processes happening in the post-cesarean uterine tissue during its healing might be causal of the CSDi instead of the niche itself. Although the pathophysiology is not fully understood yet, several factors, such as the tissue surrounding the cesarean scar with developing adenomyosis [17, 18], hemorrhaging [17], chronic inflammation [17, 18], as well as an absence of endometrium [18], are discussed as the cause of symptoms like chronical pelvic pain or dysmenorrhea. Adenomyosis is especially considered to be a potential cause of post-cesarean dysmenorrhea without having a clear pathogenesis. It is assumed that during the suturing of the uterine incision endometrial cells might be accidentally injected into the myometrium [18].

The association between dysfunctional bleeding and uterine length was an unexpected finding in our cohort. One could assume that bleeding would rather be associated with the volume of the niche instead, but this assumption is not supported by our data. Thus, our results do not correspond with preexisting studies which found an association between the size of the niche and dysfunctional bleeding [19, 20]. Other biological and genetic characteristics could be causal, and a common explanation for dysfunctional bleeding is the retention of menstrual blood and debris in the niche which the uterus can only discharge slowly due to dysfunctional myometrial contraction in the area of the scar [21]. The scar itself as a source of bleeding is supposedly another possible cause for abnormal bleeding post-cesarean. The formation of abnormal blood vessels in the area of the scar which can than cause somewhat heavy bleeding could explain the symptoms [22].

Not adjusting for the demographic characteristics of our cohort might be considered a limitation of the study, but the main aim of this work is to assess the association between the sonographic findings and clinical presentation. Therefore, adjusting for base demographic characteristics was not required. Despite its limitation, this work has several strengths. Data were collected within the context of a prospective study and the risk of information bias was low. The use of high-frequency matrix transducers and 3D ultrasound for complete visualization of the scar tissue with high-end machines is one of them. Additionally, all sonographic examinations were performed following the latest guidelines, and an adequate time gap between each CS and the examination for this study was allowed so that it is safe to assume that all our patients had fully healed scars prior to ultrasound [23].

In summary, there is no real consensus about the pathophysiology and therefore diagnostic and therapeutic options of post-cesarean gynecological complaints. Our data show some utility of ultrasound to explain dysmenorrhea with RMT as well as the position of the niche within the uterus. Nevertheless, it should be taken into consideration that further biological factors, which cannot be visualized with ultrasound, could cause those symptoms. Therefore, we are unable to solely rely on sonographic findings to help these patients and more data to support preexisting findings and help further grasp the pathophysiology are required. Future immuno-histochemical studies of the CS scars with a search for potential biomarkers present a research opportunity that should be pursued parallel to the sonographic studies.

## Data Availability

Not applicable.
